# Pretreatment of Wheat Straw with Phosphoric Acid and Hydrogen Peroxide to Simultaneously Facilitate Cellulose Digestibility and Modify Lignin as Adsorbents

**DOI:** 10.3390/biom9120844

**Published:** 2019-12-08

**Authors:** Xue Wan, Fengpei Yao, Dong Tian, Fei Shen, Jinguang Hu, Yongmei Zeng, Gang Yang, Yanzong Zhang, Shihuai Deng

**Affiliations:** 1 Institute of Ecological and Environmental Sciences, Sichuan Agricultural University, Chengdu 611130, China; wxjy1994@163.com (X.W.); yaofengpei0724@163.com (F.Y.); dongtian@sicau.edu.cn (D.T.); zengym8807@126.com (Y.Z.); gy8813@163.com (G.Y.); zyz1000@163.com (Y.Z.); shdeng8888@163.com (S.D.); 2 Chemical and Petroleum Engineering, Schulich School of Engineering, The University of Calgary, Calgary, AB T2N 4H9, Canada; jinguang.hu@ucalgary.ca

**Keywords:** lignocellulose fractionation, lignin modification, cationic substances adsorption

## Abstract

Effective valorization of lignin is crucial to achieve a sustainable, economic and competitive biorefinery of lignocellulosic biomass. In this work, an integrated process was proposed based on a concentrated phosphoric acid plus hydrogen peroxide (PHP) pretreatment to simultaneously facilitate cellulose digestibility and modify lignin as adsorbent. As a dominant constitutor of PHP pretreatment, H_2_O_2_ input and its influence on the overall fractionation/lignin modification performance was thoroughly investigated. Results indicated that wheat straw was fractionated more efficiently by increasing the H_2_O_2_ input. H_2_O_2_ input had a significant influence on the digestibility of the obtained cellulose-rich fraction whereby almost 100.0% cellulose-glucose conversion can be achieved even with only 0.88% H_2_O_2_ input. Besides, the adsorption capacity of lignin on MB was improved (74.3 to 210.1 mg g^−1^) due to the oxidative-modification in PHP pretreatment with H_2_O_2_ inputs. Regression analysis indicated that –COOH groups mainly governed the lignin adsorption (R^2^ = 0.946), which displayed the considerable adsorption capacities for typical cationic substances. This work shows a promising way to integrate the lignin modification concept into the emerging PHP pretreatment process with the dual goal of both cellulose utilization and lignin valorization.

## 1. Introduction

Fossil resources depletion and the triggered climate changes have been driving the rapid development of the bio-based economy, which aims at harvesting fuels, platform chemicals and material precursors from renewable biomass feedstocks [1,2]. Lignocellulosic biomass that mainly consists of cellulose, hemicellulose and lignin has been regarded as the most-promising and perhaps the only alternative for fossil resources to build an integrated biorefinery [3]. Lignin, the most-abundant renewable aromatic biopolymer on the Earth, is a highly branched, three-dimensional polymer derived from three phenylpropane units (monolignols), namely guaiacyl (G, coniferyl alcohol), syringyl (S, sinapyl alcohol) and *p*-hydroxyphenyl alcohol (H, *p*-coumaryl alcohol) [4,5]. Conversion of lignin into high-value products is important to increase the economic viability and environmental sustainability of the biorefinery, but this has proved challenging due to the structural complexity of lignin which depends on the biomass species, pretreatment and fractionation routes [6,7].

Lignocellulose fractionation is often performed as a primary step in the biorefinery, after which the derived major components can be converted into usable forms, and further be refined into the targeted products, such as fuels, chemicals, and materials [3,7]. Many fractionation approaches have been developed, ranging from traditional paper making (e.g., sulfite, kraft and soda) and some environmentally friendly innovations (e.g., deep eutectic solvents and ionic liquids) [6,8,9,10]. However, sulfite, kraft and soda pulping still focus on the use of readily available cellulose and hemicellulose, while the lignin by-product discharged in the form of black liquor is difficult to valorize [11]. Ionic liquids (IL) have gained much attention due to their high thermal stability, low vapor pressure, no flammability, but potential applications are restricted by high viscosity and high cost [9,10]. The emerging deep eutectic solvents (DES) are promising ionic liquid alternatives for biomass pretreatment. They usually maintain the advantages of ionic liquids but overcome their high cost and high viscosity disadvantages to some extent [10,12]. It appears that the valorization of isolated lignins obtained by prevalent pretreatments is challenging thus some post processing routes such as depolymerization and modification are still required to upgrade these lignin by-products to useful products.

Recent work has shown that lignin by-products might be a promising material for the production of various adsorbents due to its abundance in hydroxyl, methoxyl, carboxyl, and carbonyl groups [13]. These oxygen-containing functional groups could serve as bonding sites for metal ions, dyes and organic pollutants [14,15]. Although Kraft lignin is produced in great amounts by the pulping industry, it exhibits much lower chemical reactivity and functional group availability that unfortunately results in its rather low adsorption performance [5,15,16]. It was reported that lignin modification by introducing desirable functional units, such as methyl thioether, amide, sulfonic, and xanthate groups, can promote the hydrophobicity and adsorption performance [14]. It was documented that the adsorption capacity of lignin can be improved by increasing the amount of oxygen-containing functional groups on lignin, such as carboxyl and hydroxyl groups [17]. Obviously, the feasible conversion of lignin into adsorbents mainly involves the two important aspects of lignocellulose fractionation and lignin modification. However, the traditional method of separate modification after harvesting lignin will greatly complicate the process and increase the cost of lignin utilization. Therefore, integrating lignin modification into lignocellulose fractionation can be a possible way to achieve a competitive biorefinery concept [8].

As a newly developed method, the concentrated phosphoric acid plus hydrogen peroxide (PHP) pretreatment, offers some outstanding features for pretreating lignocellulosic biomass, such as high enzymatic hydrolysis efficiency, mild conditions and wide adaptability to various raw materials (such as softwood, hardwood, agricultural residues and their mixture) [18,19,20]. When PHP pretreatment was used to fractionate lignocellulosic biomass into cellulose-rich fraction, oligosaccharides and lignin, the recovered PHP lignin (PHPL) exhibited attractive properties, i.e., high purity, low molecular weight and high content of carboxylic acid groups [21,22]. Based on these investigations, it was hypothesized that PHP can work as a bifunctional pretreatment to fractionate lignocellulose, which can potentially facilitate cellulose digestibility and modify lignin to produce adsorbents. The relatively high carboxylic acid group content characteristic of the PHPL potentially allows it to be used as a promising adsorbent [17]. Since the amount of H_2_O_2_ governs the overall performance of PHP pretreatment, its influence on lignocellulose fractionation and lignin modification, as well as the adsorption performances of PHPL deserve further investigation.

In this context, the technical feasibility of integrating lignin bio-absorbents production into the emerging PHP pretreatment process was assessed. The influence of H_2_O_2_ on facilitating wheat straw fractionation and lignin modification was investigated. The chemical structural change of PHP technical lignin during the pretreatment was traced by characterization by a combination of Fourier transform infrared spectra (FT-IR) and nuclear magnetic resonance (NMR) analysis. Moreover, the typical cationic substances, including methylene blue (MB), Pb^2+^ and Cu^2+^ were employed for adsorption by the derived lignin to further clarify the adsorbent application hypothesis.

## 2. Materials and Methods

### 2.1. Feedstock

Wheat straw, as the residue of cultivated crop of wheat, has been considered an appropriate and attractive feedstock due to wide cultivation, abundance and ready biotransformation [23], thereby, it was selected for investigation in this work. It was collected from the farm of Sichuan Agricultural University, Chengdu, China. The collected wheat straw was air-dried and milled through a 40-mesh sieve for further investigation. The main chemical composition of wheat straw was determined as cellulose of 35.8% (represented by glucan content), hemicellulose of 20.6% (represented by xylan content), acid-insoluble lignin of 21.6%, acid-soluble lignin of 2.0%, and ash of 8.3%. The main chemical composition of wheat straw was determined according to Tappi-T-22 om-88 as described in reference [19]. In addition, to elucidate the structure change of lignin during PHP pretreatment with different H_2_O_2_ input, the cellulolytic enzyme lignin (CEL) isolated from wheat straw as a comparison. Main isolation procedures for CEL was according to the methods described in [24,25]. Figure S1 shows the experimental flowchart.

### 2.2. PHP-Pretreating Wheat Straw for Fractionation

Concentrated H_3_PO_4_ (85%, *w*/*w*), H_2_O_2_ (30% and 60%, *w*/*w*) and deionized water were employed for preparing the PHP mixture. In the PHP mixture, the H_3_PO_4_ proportion was 80% (*w*/*w*) and the H_2_O_2_ proportions were 0.88, 1.77 and 3.53% (*w*/*w*), respectively. In addition only 80% H_3_PO_4_ without H_2_O_2_ input was employed for a comparison. Pretreatment was carried out in a 250-mL serum bottle with 8.0 g wheat straw and 80.0 g PHP mixture with different H_2_O_2_ input. Then the sealed bottle was incubated in an orbital shaker at 50 °C for 3 h. After the pretreatment, the cellulose-rich fraction, oligosaccharides, and lignin were obtained via a fractionation process (see Figure S2), which has already established in our previous work [22]. The pretreatment of each condition was performed in duplicate, all results were recorded as the average of these two replicates. The lignin recovered from the PHP pretreatment was named PHPL. The lignin recovered from the pretreatment with 80% H_3_PO_4_ alone was named as PL.

### 2.3. Enzymatic Hydrolysis

The cellulose-rich fractions were enzymatic hydrolyzed at 2% (*w*/*v*) solid loading in sodium acetate buffer (0.05 M, pH 5.0). Cellic^®^ CTec2 (Novozymes, Beijing, China) dosage was 20 mg protein per gram cellulose. All enzymatic hydrolysis was performed in a thermostatted incubator (ZWY-2102C, Zhicheng, Shanghai, China) at 50 °C, 150 rpm. The solution (0.8 mL) was sampled at 4, 8, 12, 24, 48 and 72 h and deactivated the enzymes at 100 °C for 5 min. The supernatant was stored at −20 °C for further sugar determination.

### 2.4. Analytical Methods

The composition of raw materials, pretreated wheat straw and the obtained lignin were analyzed according to the method in the Tappi-T-22 om-88 method as described in [19]. Hydrolysate from the Klason analysis was retained and further analyzed. The acid-soluble lignin was determined by an UV spectrophotometer at 205 nm. The determined content of glucan, xylan and acid-soluble lignin acid-insoluble lignin in hydrolysate represented cellulose, hemicellulose and lignin content, respectively. The sugar moieties were measured by high-performance liquid chromatography (HPLC, Agilent 1260 Infinity system, Agilent Technologies, Inc., Santa Clara, CA, USA) equipped with a SH1011 column (Shodex, Showa Denko America, Inc., New York, NY, USA). The mobile phase was 0.05 mol L^−1^ H_2_SO_4_ pumped at a flow rate of 0.8 mL min^−1^. The column and detector were operated at 60 °C and 50 °C, respectively.

### 2.5. Lignin Characterization

The chemical functional groups on the lignin before/after MB adsorption were determined by FT-IR (Spectrum Two, PerkinElmer, Waltham, MA, USA). Lignin (4 mg) and KBr (400 mg) were homogenized in an agate mortar, and the sample was prepared using the prevalent KBr-disk method prior for the determination. Twenty scans were collected for each lignin sample with a resolution of 2 cm^−1^ over a range from 400 to 4000 cm^−1^ [2].

Quantitative ^31^P-NMR spectra of the obtained lignin were obtained according to previous work [26]. Twenty mg of lignin (dry basis) was dissolved in 0.5 mL of a mixture of anhydrous pyridine and deuterated chloroform (1.6:1, *v*/*v*) under stirring. Afterwards, 0.1 mL of 10.85 mg mL^−1^ cyclohexanol solution (dissolved in the mixture of anhydrous pyridine and deuterated chloroform, 1.6:1, *v*/*v*) as internal standard, and 0.1 mL of 5 mg mL^−1^ chromium (III) acetylacetonate (dissolved in the mixture of anhydrous pyridine and deuterated chloroform, 1.6:1, *v*/*v*) as the relaxation reagent were added the dissolved sample. Finally, 0.1 mL phosphitylating reagent (2-chloro-1,3,2-dioxaphospholane) was rapidly inputted, and the prepared sample was immediately transferred into a 5 mm tube for analysis on NMR (AVII 600, Bruker, Karlsruhe, Germany).

2D heteronuclear single quantum coherence nuclear magnetic resonance (HSQC NMR) spectra were acquired on a Bruker AVIII 600 MHz spectrometer. Lignin (100 mg, dry basis) was dissolved in 0.8 mL of DMSO-d_6_. Chromium (III) acetylacetonate (10 μmol mL^−1^) was added to the mixture to provide complete relaxation of all nuclei, and the mixture was immediately transferred into a 5 mm tube for analysis. The spectral widths were 5000 and 20,000 Hz for the ^1^H and ^13^C dimensions, respectively. The number of collected complex points was 1024 for the ^1^H dimension with a recycle delay of 1.5 s. The number of transients was 64 and 256-time increments were recorded in the ^13^C dimension. The central solvent (DMSO) peak (δC/δH 39.5/2.49) was used as an internal chemical shift reference point. Cross peaks were assigned by comparison with the literature using MestReNova 9.0.1 (Mestrelab Research, Santiago de Compostela, Spain) [27].

### 2.6. Adsorption of Cationic Substances by PHPLs

Isothermal adsorption tests were performed to determine the maximum adsorption capacity for MB. Isolated lignin (100 mg) was mixed with 50 mL solution in 100 mL conical flask containing MB with different initial concentrations of 20, 50, 100, 200, 400, 800, and 1200 mg L^−1^, respectively. The conical flask was sealed and shaken in a thermostat incubator (ZWY-2102C, Zhicheng, Shanghai, China) with shaking frequency of 150 r min^−1^ at 25 °C for 24.0 h. The samples were filtered through a 0.45 μm nylon filter for determining the MB concentration using an UV visible spectrophotometer (EU-2600A, Onlab, Shanghai, China) at 664 nm. The adsorption capacity was calculated by Equation (1):

(1) $$Q_{e}{\;=\;(C}_{o}{\;-\;C}_{e})V/m$$

where C_o_ (mg L^−1^) and C_e_ (mg L^−1^) are the initial and equilibrium concentrations of MB, respectively. V (L) is the volume of the solution, and m (g) is the mass of lignin.

For interpretation of the interaction between the lignin and MB, Langmuir, and Freundlich adsorption isotherm models were employed to analyze experimental data and describe the equilibrium of adsorption. The Langmuir and Freundlich equations are represented as Equation (2) and Equation (3) [13]:

(2) $$Q_{e\;}{=\;Q}_{\max}K_{L}C_{e}{/(1\;+\;K}_{L}C_{e})$$

(3) $$Q_{e}=K_{F}C_{e}^{1/n}$$

where Q_e_ (mg g^−1^) and C_e_ (mg L^−1^) are the amount adsorbed of MB at equilibrium and the adsorbate concentration in solution. Q_max_ (mg g^−1^) and K_L_ (mL g^−1^) are the Langmuir constants related to the saturated sorption capacity and sorption energy, respectively. K_F_ [(mg g^−1^) (L mg^−1^)1/n] is Freundlich constant, indicating the capacity of the adsorption, and n is the heterogeneity factor.

Lignin (100 mg) was mixed with 50 mL solution with a concentration of 400 mg L^−1^ of MB for the assessment of adsorption kinetics at 25 °C. 0.5 mL solution was withdrawn at 0.25, 0.5, 0.75, 1, 1.5, 2, 4, 8, 12, 24 and 48 h for the concentration determination. The pseudo-first-order Equation (4) and pseudo-second-order Equation (5) models were used to assess the adsorption kinetics [13]:

(4) $$\mathrm{Log}\left(Q_{e}{\;-\;Q}_{t}\right){\;=\;\mathrm{Log}\;Q}_{e}{\;-\;K}_{1}t/2.303$$

(5) $${t/Q}_{t}{\;=\;1/K}_{2}Q_{e\;}^{2}{+\;t/Q}_{e}$$

where t is the response duration (h) of MB in the aqueous solution. Q_e_ is the adsorption capacity (mg g^−1^) at equilibrium time. Q_t_ is the adsorption capacity (mg g^−1^) at the timepoint of t, and K_1_ and K_2_ are the rate constants of pseudo-first-order model and pseudo-second-order model, respectively.

Besides, Pb^2+^ and Cu^2+^, as another typical cationic substance, were used to determine the adsorption capacity of PHPL. PHPL (100 mg) was mixed with 50 mL Pb^2+^ and Cu^2+^ aqueous solution with the concentration of 200 mg L^−1^. The detailed adsorption protocols were similar to those of MB. The batch removal was performed in duplicate, and all results were recorded as the average of these two replicates. After adsorption, the lignin was immediately filtered and the residual metal ion concentration in the filtrate was determined by a flame atomic absorption spectrophotometry (FAAS-M6, Thermo Fisher, Waltham, MA, USA).

## 3. Results and Discussion

### 3.1. Wheat Straw Fractionation via PHP Pretreatment

**Figure 1:**
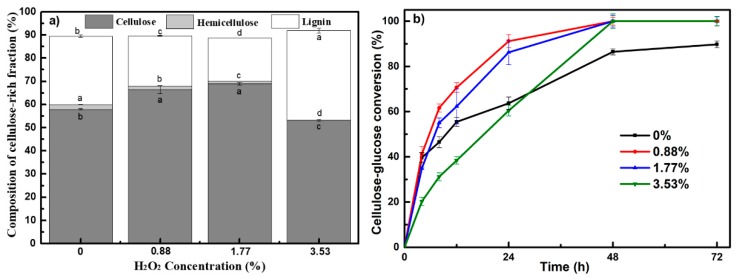
Main composition and enzyme hydrolysis of cellulose-rich fraction resulting from various H_2_O_2_ input during PHP pretreatment. (**a**) Main chemical composition; (**b**) Performances of enzyme hydrolysis (the employed solid loading for hydrolysis was 2%, CTec2 dosage was 20 mg protein per gram cellulose).

As shown in Table 1, the input of H_2_O_2_ significantly influenced the yield of cellulose-rich fraction (*p* < 0.05). A 45.8 g to 38.2 g range of cellulose-rich fractions were obtained when the H_2_O_2_ proportion increased from 0.88% to 1.77%, in comparison to 53.7 g without H_2_O_2_ input. Although the yield of cellulose-rich fraction decreased, the cellulose content in the recovered solid fraction was significantly increased from 57.9% to 68.9% (Figure 1a), and the hemicellulose and lignin content were significantly reduced. This indicated that the addition of H_2_O_2_ in PHP pretreatment can promote lignin and hemicellulose removal (see Table S1), thus improving the purity of cellulose. When the cellulose-rich fraction was employed for enzymatic hydrolysis, it was shown PHP pretreatment produced a much more digestible cellulose-rich fraction than single H_3_PO_4_ pretreatment (see Figure 1b). It was shown H_2_O_2_ addition significantly enhanced cellulose-glucose conversion in that almost 100% digestibility was achieved even with only 0.88% addition. It was interesting that although 3.53% H_2_O_2_ addition compromised cellulose recovery (only 24.2% recovery), 100% cellulose-glucose conversion still occurred. As reported, the residual lignin in the pretreated solid generally adsorbs cellulases via hydrophobic interactions, electrostatic, and hydrogen bonding interactions, resulting effects on enzymatic hydrolysis [28]. Therefore, it will be deserved some substantial investigations to clarify the characteristics of residual lignin [29], and further explain the relationship between lignin and enzymatic hydrolysis in future work.

The input of H_2_O_2_ significantly influenced the yield of lignin (*p* < 0.05). From 7.1 g to 7.7 g of derived lignin was obtained when the H_2_O_2_ proportion increased from 0.88% to 3.53%, in comparison to 5.1 g without H_2_O_2_ input. The pretreatment without H_2_O_2_ involvement (only 80% H_3_PO_4_) showed a limited ability to fractionate wheat straw. This was greatly related to the ability of H_3_PO_4_, which could dissolve cellulose and hemicellulose by breaking up the ordered hydrogen bonds among sugar chains to achieve the fractionation [30]. However, the separation efficiency was improved with H_2_O_2_ input. It was suggested that the oxidation of H_2_O_2_ could promote the separation function of H_3_PO_4_. As for oligosaccharide, the input of H_2_O_2_ had no significantly influence (*p* > 0.05) on its yield when the H_2_O_2_ input was increased from 0.88% to 1.77%, in comparison to that of 5.8 g without H_2_O_2_ input. However, when 3.53% H_2_O_2_ was used, the yield of oligosaccharide and cellulose-rich fraction decreased significantly to 0.8 g and 16.3 g (*p* < 0.05), respectively. This was due to the cellulose and oligosaccharide degradation caused by an excessive amount of H_2_O_2_. These carbohydrates might be degraded by HO^+^ or O· and OH· that were formed with higher H_2_O_2_ input to form furfural, acetic acid and formic acid [21]. The addition of H_2_O_2_ in amounts greater than 1.77% was not conducive to the recovery of cellulose-rich fractions and oligosaccharides. Thus, further optimizing H_2_O_2_ input in PHP pretreatment is required to construct an emerging integrated biorefinery of lignocellulosic biomass.

### 3.2. Lignin Modification During PHP Pretreatment

To better elucidate the effects of H_2_O_2_ input on lignin properties in PHP modification, a combination of several techniques (e.g., HPLC, FT-IR and NMR) was employed in this study. When the residual carbohydrates content in the resultant lignin was determined by the HPLC (see Table 2), PHPLs exhibit a rather high purity than the lignin recovered from H_3_PO_4_ alone pretreatment (PL). A higher purity of PHPL was observed with more H_2_O_2_ input, especially when the H_2_O_2_ input was great than 1.77%. When only H_3_PO_4_ was employed to fractionate wheat straw, only 23.7% lignin was recovered in the solid fraction. However, 32.8% of PHPL was harvested at the lowest H_2_O_2_ input of 0.88% in PHP pretreatment, whereas it was significantly increased to 35.7% at the highest H_2_O_2_ input of 3.53% (*p* < 0.05). This phenomenon indicated that the addition of H_2_O_2_ as an oxidant promoted the oxidative depolymerization of lignin for the subsequent recovery [7]. Comparing the GPC data of PHPL-1.77% and CEL, it could be found that PHPL-1.77% had a low molecular weight of about 1436 g mol^−1^ (see Figure S3). This result additional evidence that lignin was oxidatively degraded by the H_2_O_2_ input in PHP pretreatment.

FT-IR analysis was carried out to acquire some basic information about the chemical structure of these lignins (see Figure S4). As expected, the spectra of these four lignins were quite different. Some typical aromatic ring absorption bands at 1034 cm^−1^ (C–H in-plane deformations in G units), 1126 cm^−1^ (C–H in-plane of S units), 1422 cm^−1^ (C–H in-plane deformation with aromatic ring stretching), and 1509 cm^−1^ (C=C stretching of aromatic ring) were observed for PL [31,32]. However, the intensity of those bands was dramatically decreased in PHPLs with more addition of H_2_O_2_, indicating the aromatic units might be destroyed. In addition, the band at 1730 cm^−1^, originated from C=O stretching in unconjugated ketones or carboxyl groups, was greatly increased in PHPLs compared with PL, also potentially suggesting the substantial lignin oxidation happened in PHP [33,34].

Actually, the H_2_O_2_ in the PHP pretreatment was responsible for releasing hydroxyl cations (HO^+^) or free radicals, such as O·, OH·, which was mainly related to lignin removal by oxidation, as well as the possible modification on the recovered lignin [7,21,22]. To further elucidate the effects of H_2_O_2_ input on lignin modification, the ^31^P-NMR technique was applied to quantify the content of carboxylic hydroxyl groups, phenolic hydroxyl groups and aliphatic hydroxyl groups [35]. The comparative ^31^P-NMR spectra are shown in Figure S5, and the proportions of functional groups calculated from the ^31^P-NMR are summarized in Table 3.

As a representative source of native lignin, cellulolytic enzyme lignin (CEL) from wheat straw was also employed for comparison. The aliphatic hydroxyl groups, carboxylic hydroxyl groups and total phenolic hydroxyl groups on CEL were determined as 4.81, 0.15 and 1.40 mmol g^−1^, respectively. As for aliphatic –OH, its content did not exhibit obvious changes as the H_2_O_2_ input was increased from 0.88% to 1.77% compared with PL. However, it was greatly reduced compared to that of CEL, suggesting that both PL and PHPL were dehydrated under the strong acidic conditions of the concentrated H_3_PO_4_ [36]. The total phenolic –OH on PL were increased compared with CEL, suggesting the cleavage of β-O-4′ linkages by acidic depolymerization in the concentrated H_3_PO_4_ [27]. In addition, total phenolic –OH on PHPL were significantly increased as the H_2_O_2_ input increased, suggesting β-O-4′ linkages can be cleaved by oxidation. However, a seriously decrease of the aliphatic –OH and the total phenolic –OH content appeared at 3.53% H_2_O_2_ input. The aliphatic –OH was reduced from 1.79 mmol g^−1^ (at 1.77% H_2_O_2_ input) to 1.54 mmol g^−1^ (at 3.53% H_2_O_2_ input), and the total phenolic –OH was reduced from 2.54 mmol g^−1^ (at 1.77% H_2_O_2_ input) to 2.38 mmol g^−1^ (at 3.53% H_2_O_2_ input). These facts indicated that further oxidation potentially occurred with more H_2_O_2_ input. Furthermore, the carboxylic –OH content in PHPL was significantly promoted from 0.66 mmol g^−1^ to 0.76 mmol g^−1^ (*p* < 0.05) compared with PL as the H_2_O_2_ input increased from 0.88% to 3.53%. Obviously, the oxidative depolymerization was intensified by increasing H_2_O_2_ input, which can cleave more β-O-4′ linkages or other side-chains, releasing more carboxylic -OH groups consequently [37]. 

**Figure 2:**
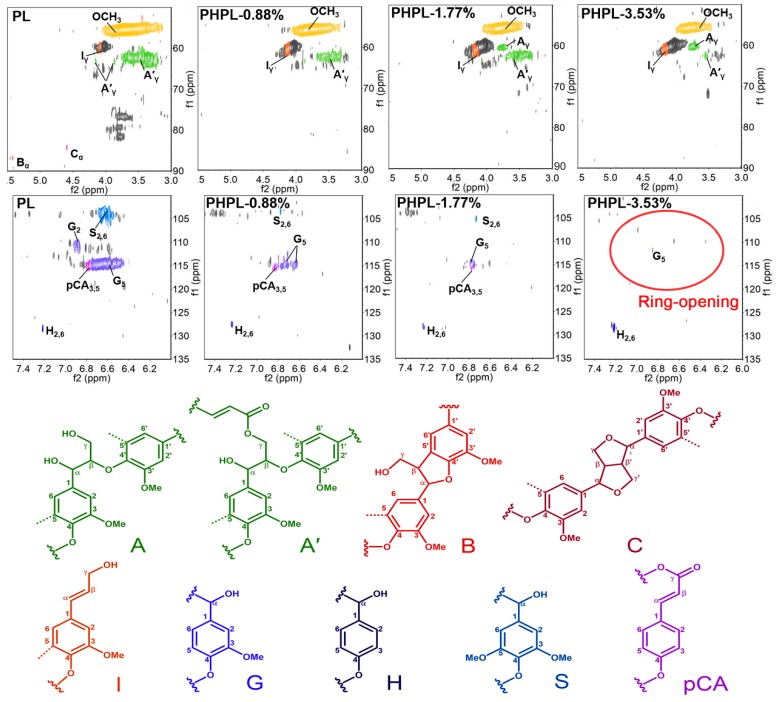
2D-HSQC NMR spectra of PL and PHPLs. Side-chain linkages: (A) β-O-4′ structures, (A′) γ-acylated β-O-4′ substructures, (B) β-5′ phenylcoumaran substructures, (C) β-β′ resinol substructures. Aromatic units: (G) guaiacyl units, (H) *p*-hydroxyphenyl units, (S) syringyl units, (pCA) *p*-coumarate. The uncolored cross peaks are signals of sugars or unidentified lignin substructures.

Besides, the main substructures and their linkages in PL and PHPLs were subsequently assessed using 2D-HSQC NMR (see Figure 2 and Table S2). The 2D HSQC NMR spectra of lignins show three regions corresponding to aliphatic (δ_C_/δ_H_: 10−50/0.5−3.0), side-chain (δ_C_/δ_H_: 50–90/2.5–6.0) and aromatic (δ_C_/δ_H_: 90–135/5.5–8.5) ^13^C–^1^H correlation. In the side-chain region, methoxyl (δ_C_/δ_H_: 55.6/3.73) and β-O-4′ substructures A′_γ_ (δ_C_/δ_H_: 63.1/3.83–4.30) were the most prominent signals in PL. However, the correlation signals of A′_γ_ decreased, especially with the 3.53% H_2_O_2_ input. It again proved the cleavage of β-O-4′ linkages in PHP pretreatment. In the aromatic region, signal from H_2,6_ (δ_C_/δ_H_: 127.8/7.19), S_2,6_ (δ_C_/δ_H_: 103.8/6.69) and G_2_ (δ_C_/δ_H_: 110.9/6.99) were observed in the spectra of PL, confirmed that the lignin in wheat straw was GSH-type [33]. However, the correlation signals for S_2,6_, G_2_ and pCA substructures were absent after PHP pretreatment, especially almost no S_2,6_ and G_2_ signal in the PHPL-3.53%. 

It was suggested that the ring-opening oxidation on those aromatic units was intensified with the increased of H_2_O_2_ input, and the content of carboxylic –OH was increased accordingly [21,22]. These results further explained the reason for the decreasing of typical aromatic ring absorption bands in FT-IR spectra. Therefore, these results proved that PHP pretreatment can achieve lignin modification through β-O-4′ linkages cleavage and ring-opening oxidation. The degree of modification on lignin can be control by changing the addition of H_2_O_2_ input. The resultant PHP lignin has abundant oxygen-containing functional groups, and can potentially work as a functional adsorbent to remove some cationic substances from aqueous solution [17,38].

### 3.3. Adsorption of Cationic Substances by PHPLs

**Figure 3:**
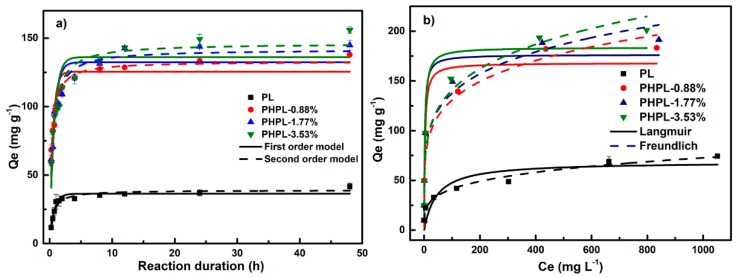
Adsorption behavior of MB onto these four lignins. (**a**) Kinetics; (**b**) Isotherms.

It is well known that hydroxyl and carboxyl acid groups as the surface functional groups on lignin can bond metal ions, dyes and organics with the lignin via electrostatic attraction, ion-exchange and complexation [39]. To assess the potential of adsorption ability on cationic substances by PHPLs, MB, as a typical cationic dye, was firstly selected here as a model compound [40]. The pseudo-first-order and pseudo-second-order kinetic model and the Langmuir and Freundlich isotherm models were employed to investigate the MB adsorption behaviors by the harvested lignins (see Figure 3) [13].

Based on the kinetic simulation, the pseudo-second order kinetic model displayed a better fit than that of the pseudo-first-order model for PL (see Figure 3a), PHPL-0.88%, PHPL-1.77% and PHPL-3.53% with relatively higher R^2^ of 0.994, 0.960, 0.944 and 0.937, respectively (see Table S3). It was suggested that the MB adsorption process by PL and PHPLs was controlled by a chemisorption mechanism [13,40]. Besides, the calculated value of Q_e_ from the pseudo-second-order kinetic model were very close to the experimental adsorption capacities (Q_ex_), again proving the pseudo-second order kinetic model can describe the MB adsorption better. Besides, the Freundlich isotherm exhibited a better correlation coefficient than Langmuir isotherm for PL (see Figure 3b), PHPL-0.88%, PHPL-1.77% and PHPL-3.53% with R^2^ of 0.982, 0.950, 0.933 and 0.942, respectively (see Table S4). This indicated that the MB adsorption by PL and PHPLs occurred at multilayer and heterogeneous surface [41]. According to the experimental results, the MB adsorption capacities by the as-obtained lignins followed the order of PHPL-3.53% (201.1 mg g^−1^) > PHPL-1.77% (188.3 mg g^−1^) > PHPL-0.88% (183.2 mg g^−1^) > PL (74.3 mg g^−1^). Obviously, PHPLs exhibited greater adsorption capacity than PL, which was probably due to a relatively higher oxygen-containing functional groups content on PHPLs via oxidative modification. By contrast, MB adsorption capacities of lignins and lignin-based materials were 31.2–80.6 mg g^−1^ (see Table 4) [40,42,43,44,45]. Thus, the excellent adsorption capacity of PHPLs made them in application more feasible.

**Figure 4:**
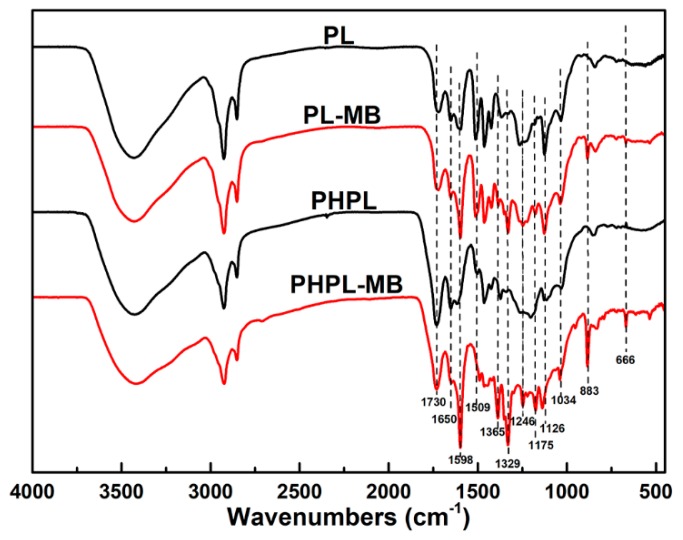
FT-IR spectra of lignin before/after MB adsorption. PL-MB and PHPL-MB refer to the lignin after MB adsorption.

When PHPLs before/after adsorbing MB were observed under SEM (see Figure S6), it appeared that the solid particles congregated on the surface of PHPL, and transformed into a compact and flat surface after adsorption. This change in the surface morphology can be deduced to be due to the bonded MB on lignin. As shown in Figure 4, the peak at 3425 cm^−1^ (–OH) was shifted to 3418 cm^−1^, and the signal at 1730 cm^−1^ (C=O stretching in unconjugated ketones or carboxyl groups) was decreased after adsorption [34]. Meanwhile, the band at 1598 cm^−1^, representing the –COO^−^ stretching vibration, became stronger after adsorption [46]. These changes indicated that –OH and –COOH were involved in the adsorption process [46,47]. Generally, the -OH of lignin linkages with cations groups through electrostatic attractions or surface exchanges, while the –COOH groups were ionized as –COO^−^ groups at higher pH, and provided potential sites for coordinative linkages with cations groups [38,40,48]. Based on these results, the enhanced adsorption capacity of PHPLs on MB can be greatly attributed to the increased –OH and –COOH on the surfaces of PHPLs, which was mainly resulted from the oxidative modification in the pretreatment.

**Figure 5:**
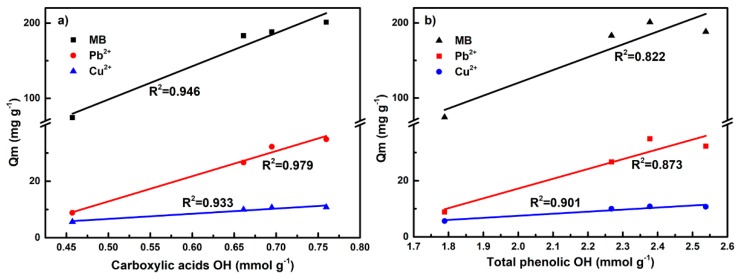
Correlation analysis of the content hydroxyl groups and maximum sorption capacities, (**a**) Carboxylic acids OH; (**b**) Total phenolic OH.

According to the analysis above, it seemed the oxygen-containing groups (e.g., Ar–OH, and –COOH) provided electron donating sites for MB adsorption. Moreover, the maximum adsorption capacity of PHPLs was increased with the content increase of carboxylic –OH and phenolic –OH. A further correlation analysis indicated that the adsorption capacity of lignin exhibited a good line relationship with the content of phenolic –OH, and –COOH (see Figure 5). Moreover, the linear fitting of carboxyl group with adsorption capacity (R^2^ = 0.946) was better than that of total phenol hydroxyl group (R^2^ = 0.822), suggesting that the carboxylic of the lignin surface played a dominant role in the MB adsorption. Actually, a similar report has indicated the –COOH, usually as the main functional groups, was introduced on lignin and other carriers by additional modification to improve adsorption capability [17,38,41]. Apparently, PHP pretreatment demonstrated the advantage of producing PHPLs with the key functional –COOH groups by a one-step fractionation and modification compared with other methods [17,38].

In order to further verify the adsorption function of lignin modification by PHP pretreatment, typical cationic heavy metal substances (Pb^2+^ and Cu^2+^) were also tested for adsorption investigation. Basically, almost similar results can be achieved, in which PHPLs exhibited greater adsorption capacity than PL. Moreover, the adsorption capacity by PHPLs on Pb^2+^ and Cu^2+^ still displayed a positive correlation with the content of total phenolic –OH, and –COOH (see Figure 5). The correlation coefficients of the linear fitting between carboxyl groups and adsorption capacity were 0.979 and 0.933 for Pb^2+^ and Cu^2+^, respectively, which was better than the total phenolic –OH, also suggesting the curial function of lignin –COOH on adsorbing cationic substances.

Overall, the synergism from the concentrated H_3_PO_4_ and H_2_O_2_ can achieve a dual function to enable an efficient fractionation of lignocellulose and simultaneously harvest the oxidatively modified-lignin. The adsorption capacity of resultant lignin was significantly enhanced, even with only 0.88% H_2_O_2_ input. Taking into the adsorption performance and fractionation into consideration, it appeared that 0.88–1.77% H_2_O_2_ input gave the best compromise between cellulose digestibility and lignin modification for subsequent adsorption applications.

## 4. Conclusions

PHP, which can simultaneously facilitate cellulose digestibility and modify lignin as adsorbents, was successfully used for fractionating wheat straw. The fractionation efficiency for wheat straw was improved with increasing the H_2_O_2_ input in the pretreatment, and the removal of recalcitrant hemicellulose and lignin fraction were promoted correspondingly. The obtained cellulose-rich fraction displayed high digestibility for saccharification. Lignin was modified by β-O-4′ linkages cleavage and ring-opening oxidation, which featured by abundant oxygen-containing functional groups. The adsorption capacity of resultant lignin was significantly enhanced due to the amount of –COOH groups formed. This proposed route might be extended to other pretreatment systems or biomass species to produce other value-added lignin adsorbents.

## Figures and Tables

**Table 1:** Recovery of cellulose-rich solids, lignin and oligosaccharide fraction form 100g wheat straw (dry basis) at various H_2_O_2_ input based on the fractionation process.

H_2_O_2_ Input	0%	0.88%	1.77%	3.53%
Cellulose-rich solids (g)	53.7 ± 0.38 ^a^	45.8 ± 0.29 ^b^	38.2 ± 0.07 ^c^	16.3 ± 0.59 ^d^
Oligosaccharide (g)	5.8 ± 0.03 ^a^	4.5 ± 0.01 ^a^	5.1 ± 0.01 ^a^	0.8 ± 0.09 ^b^
PHP lignin (g)	5.1 ± 0.48 ^d^	7.1 ± 0.55 ^c^	7.5 ± 0.01 ^b^	7.7 ± 0.05 ^a^

Note: the different lowercase for each row in this table means the difference is significant (*p* < 0.05) among the yield of recycled products at various H_2_O_2_ input.

**Table 2:** Yield and residual carbohydrates content in the recovered lignins.

Samples	Yield (%)	Carbohydrates (%)		
		Glucose	Xylose	Total
PL ^#^	23.7 ± 0.16 ^d^	0.49 ± 0.00 ^b^	0.85 ± 0.16 ^a^	1.33 ± 0.01 ^a^
PHPL-0.88%	32.8 ± 0.06 ^c^	0.30 ± 0.05 ^c^	0.76 ± 0.16 ^a^	1.06 ± 0.08 ^b^
PHPL-1.77%	34.8 ± 0.05 ^b^	0.20 ± 0.02 ^d^	0.53 ± 0.16 ^b^	0.74 ± 0.06 ^c^
PHPL-3.53%	35.7 ± 0.41 ^a^	0.72 ± 0.00 ^a^	0.16 ± 0.16 ^c^	0.87 ± 0.04 ^c^

# PL refers to the lignin which obtained by 80% H_3_PO_4_-pretreatment. PHPL-0.88%, PHPL-1.77% and PHPL-3.53% refer to the lignin which obtained by PHP pretreatment under 0.88%, 1.77% and 3.53% H_2_O_2_ input, respectively. The different lowercase letters in each column indicated significant differences (*p* < 0.05) among yield and carbohydrates content in the different lignin.

**Table 3:** Contents (mmol g^−1^) and locations of hydroxyl groups in the recovered lignin as quantitatively determined by ^31^P NMR spectroscopy.

Samples	CEL	PL	PHPL-0.88%	PHPL-1.77%	PHPL-3.53%
Aliphatic OH	4.81	1.73	1.76	1.79	1.54
C-5 substitution	0.35	0.87	1.17	1.39	1.36
Guaiacyl phenolic OH	0.60	0.61	0.68	0.70	0.62
*p*-Hydroxyphenyl OH	0.46	0.30	0.42	0.45	0.39
Carboxylic acids OH	0.15	0.46	0.66	0.69	0.76
Total phenolic OH	1.40	1.79	2.27	2.54	2.38

**Table 4:** Comparison of MB adsorption capacities for various lignin-based adsorbents.

Adsorbent	T (K)	Adsorption Capacity Q_max_ (mg g^−1^)	Refs.
PHPL	298	201.1	This study
Natural lignin	a	80.6	[42]
Organosolv lignin	293	40.0	[43]
Lignin-chitosan blended extrudates	293	36.3	[40]
Modified lignin	323	34.2	[44]
Lignin-based hollow microsphere	313	31.2	[45]

a Room temperature.

## Supplementary Materials

The following are available online at https://www.mdpi.com/2218-273X/9/12/844/s1. Figure S1: Isolation procedure for cellulolytic enzyme lignin, Figure S2: Flowchart of fractionating wheat straw by PHP pretreatment, Figure S3: Weight-average (Mw) and number-average (Mn) molecular weights (g mol^−1^) and polydispersity (Mw/Mn) of CEL and PHP lignin, Figure S4: FTIR spectra of lignins, Figure S5: Quantitative ^31^P NMR spectra of CEL and three PHP lignins, Figure S6: SEM images of lignin before/after adsorbing MB, Table S1: Responses of H_2_O_2_ concentration on pretreatment efficiency according to the hemicellulose, lignin removal and cellulose recovery, Table S2: Assignments of ^13^C–^1^H Correlation Signals in the 2D-HSQC Spectra of the CEL and three PHP Lignins, Table S3: Parameters of MB adsorption kinetics, Table S4: Parameters of Langmuir and Freundlich model for MB adsorption by recovered lignins.
