# Supplementary material for: Pretreatment of Wheat Straw with Phosphoric Acid and Hydrogen Peroxide to Simultaneously Facilitate Cellulose Digestibility and Modify Lignin as Adsorbents
Source: Biomolecules. 2019 Dec 8;9(12):844. doi: 10.3390/biom9120844 (PMC6995591; doi:10.3390/biom9120844)
Supplement: Supplementary file 1 [file biomolecules-09-00844-s001.pdf]

biomolecules

MDPI

Supporting information for

Pretreatment of Wheat Straw with Phosphoric Acid and Hydrogen Peroxide to Simultaneously Facilitate Cellulose Digestibility and Modify Lignin as Adsorbents

Xue Wan [1], Fengpei Yao [1], Dong Tian [1], Fei Shen [1,*], Jinguang Hu [2], Yongmei Zeng [1], Gang Yang [1], Yanzong Zhang [1] and Shihuai Deng [1]

1 Institute of Ecological and Environmental Sciences, Sichuan Agricultural University, Chengdu, Sichuan 611130, China; wxjy1994@163.com (X.W.); yaofengpei0724@163.com (F.Y.); dongtian@sicau.edu.cn (D.T.); zengym8807@126.com (Y.Z.); gy8813@163.com (G.Y.); zyz1000@163.com (Y.Z.); shdeng8888@163.com (S.D.)

2 Chemical and Petroleum Engineering, Schulich School of Engineering, The University of Calgary, Calgary, AB T2N 4H9, Canada; jinguang.hu@ucalgary.ca

* Correspondence: fishensjtu@gmail.com or fishen@sicau.edu.cn; Tel.: +86-28-86293087

Experimental Section

Lignin characterization

The weight-average (Mw) and number-average (Mn) of CEL and PHPL-1.77% were measured by the gel permeation chromatography (GPC) (Agilent 1200, Agilent Technologies, Inc., USA) to preliminarily confirm the degree of depolymerization with H_2O_2 input. 0.2 g lignin was dissolved in 3.0 mL of pyridine, and 3.0 mL of acetic anhydride was subsequently added for the acetylation before GPC determination [1].

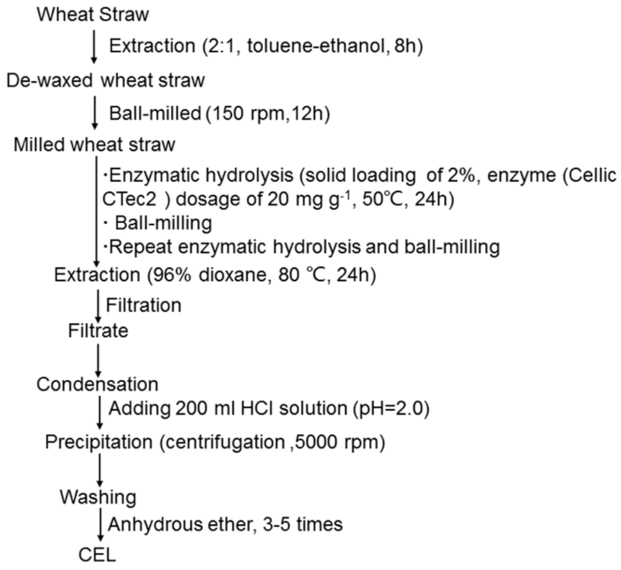

Figure S1. Isolation procedure for cellulolytic enzyme lignin.

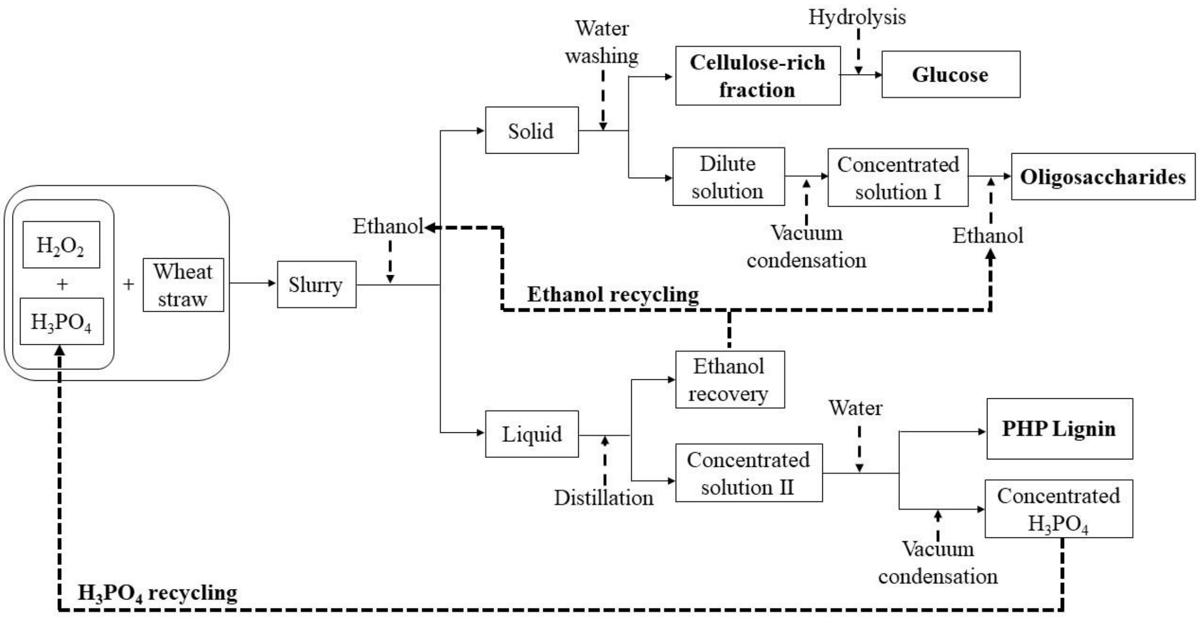

Figure S2. Flowchart of fractionating wheat straw by PHP pretreatment.

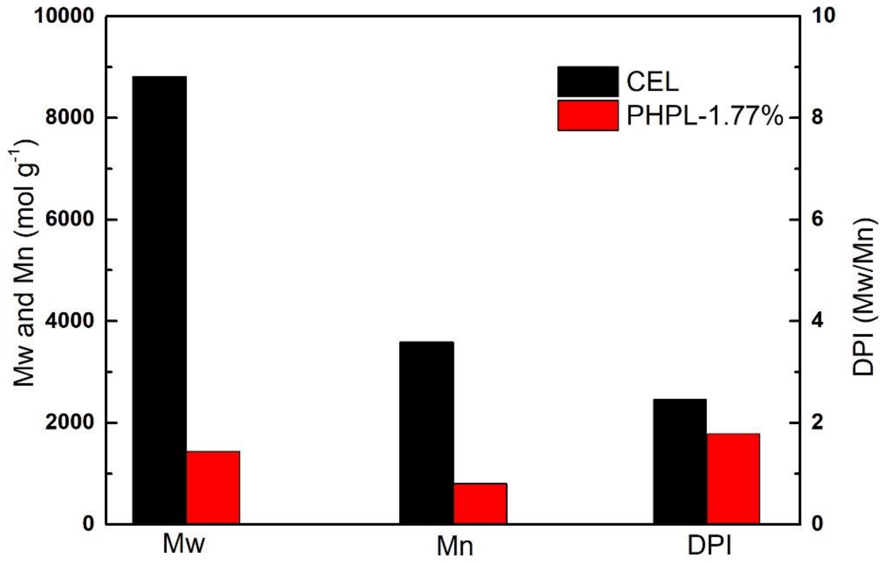

Figure S3. Weight-average (M_w) and number-average (M_n) molecular weights (g mol^{-1}) and polydispersity (M_w/M_n) of CEL and PHP lignin.

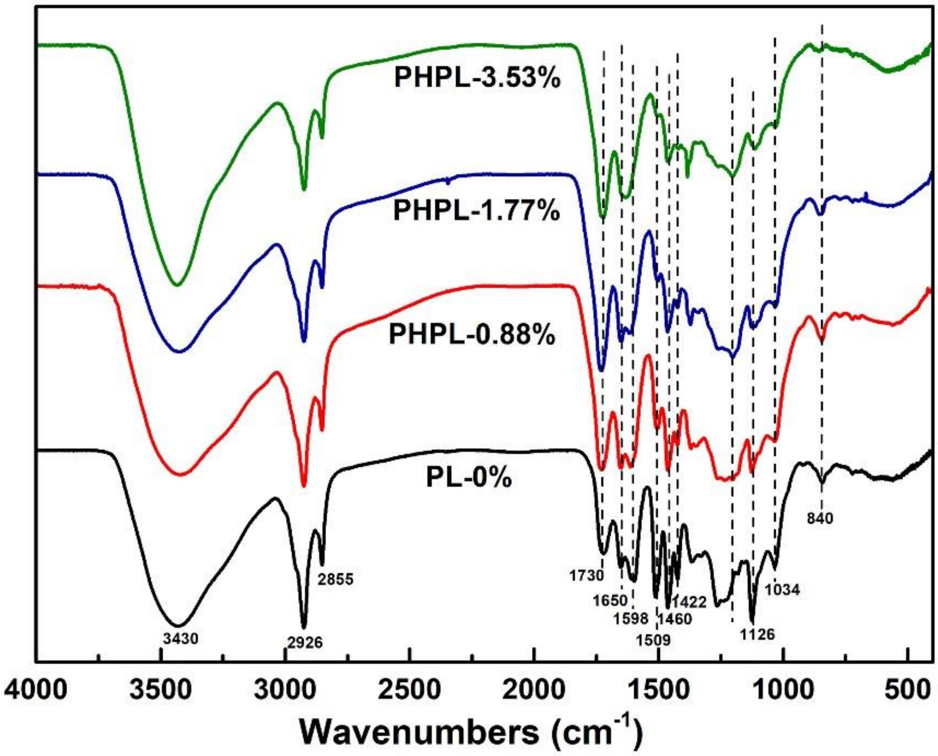

Figure S4. FTIR spectra of lignins.

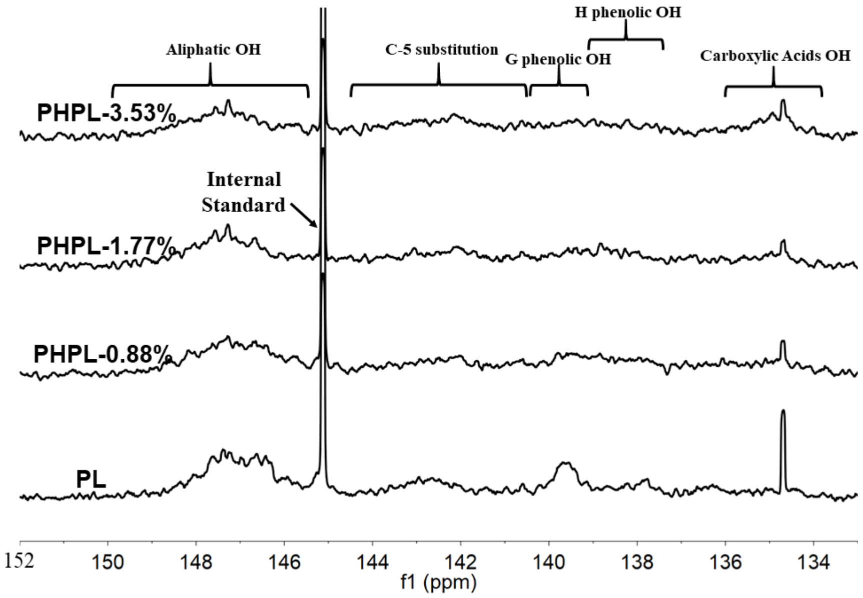

Figure S5. Quantitative ^{31}P NMR spectra of CEL and three PHP lignins.

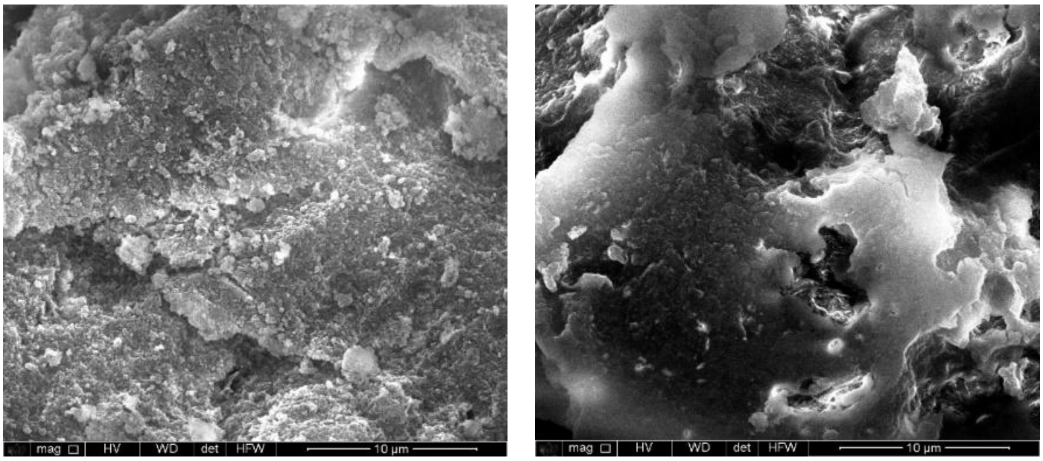

Figure S6. SEM images of lignin before/after adsorbing MB.

Table S1. Responses of H_2O_2 concentration on pretreatment efficiency according to the hemicellulose, lignin removal and cellulose recovery.

**H_2O_2 input**	**0%**	**0.88%**	**1.77%**	**3.53%**
Cellulose recovery (%)	86.8±1.12[a]	85.0±1.72[a]	73.5±0.69[b]	24.2±1.05[c]
Hemicellulose removal (%)	94.5±0.09[d]	96.4±0.02[c]	97.8±0.12[b]	100.0±0.00[a]
Lignin removal (%)	32.7±1.57[d]	57.8±0.65[c]	69.8±0.06[b]	73.3±1.75[a]

Note: the different lowercase for each row in this table means the difference is significant ($p<0.05$)

Table S2. Assignments of ^{13}C−1H Correlation Signals in the 2D-HSQC Spectra of the CEL and three PHP Lignins.

Label	δ_C/δ_H (ppm)	assignment
B_β	53.5/3.46	C_β-H_β in β-5′ phenylcoumaran substructures (B)
$-OCH_3$	55.6/3.73	C-H in methoxyls
A_γ	60.0/3.38-3.71	C_γ–H_γ in γ-hydroxylated β-O-4′ substructures (A)
I_γ	61.3/4.09	C_γ-H_γ in cinnamyl alcohol end-groups (I)
A'_γ	63.1/3.83-4.30	C_γ-H_γ in γ-acylated β-O-4′ substructures (A′)
C_α	84.8/4.67	C_α-H_α in β-β′ resinol substructures (C)
$S_{2,6}$	103.8/6.69	C_2-H_2 and C_6-H_6 in etherified syringyl units (S)
G_2	110.9/6.99	C_2-H_2 in guaiacyl units (G)
G_5/G_6	114.9/6.72 and 6.94, 118.9/6.77	C_5-H_5 and C_6-H_6 in guaiacyl units (G)
$PCA_{3,5}$	115.5/6.77	C_3-H_3 and C_5-H_5 in p-coumarate (PCA)
$H_{2,6}$	127.8/7.19	$C_{2,6}$-$H_{2,6}$ in p-hydroxyphenyl units (H)

Table S3. Parameters of MB adsorption kinetics.

Lignin	Q_{ex} (mg/g)	Pseudo-first-order model			Pseudo-second-order model		
		Q_e (mg/g)	K_1 (1/h)	R^2	Q_e (mg/g)	K_2 (mg/(g·h))	R^2
PL	41.6	36.3	1.457	0.923	39.0	0.055	0.994
PHPL-0.88%	137.9	125.5	1.952	0.796	133.0	0.025	0.960
PHPL-1.77%	144.9	132.4	1.439	0.794	141.7	0.016	0.944
PHPL-3.53%	155.9	136.2	1.419	0.772	146.2	0.014	0.937

Table S4. Parameters of Langmuir and Freundlich model for MB adsorption by recovered lignins.

Lignin	Langmuir			Freundlich		
	Q_m (mg g^{-1})	K_1	R^2	K_2	n	R^2
PL	68.60	0.022	0.782	13.500	4.105	0.982
PHPL-0.88%	168.03	0.346	0.911	54.373	5.243	0.950
PHPL-1.77%	176.35	0.443	0.924	57.562	5.277	0.933
PHPL-3.53%	183.68	0.338	0.911	55.447	4.935	0.942

1. Alvarez-Vasco, C.; Ma, R.; Quintero, M.; Guo, M.; Geleynse, S.; Ramasamy, K.K.; Wolcott, M.; Zhang, X. Unique low-molecular-weight lignin with high purity extracted from wood by deep eutectic solvents (DES): A source of lignin for valorization. *Green Chem.* **2016**, *18*, 5133–5141.
